# Pilocarpus in Erysipelas

**Published:** 1888-09

**Authors:** W. C. C. Stirling

**Affiliations:** White Oak, Texas


### PILOCARPUS IN ERYSIPELAS.

BY W. C. C. STIRLING, M. D., WHITE OAK, TEXAS.

I noticed in the *Medical World* that Professor Waugh, of Philadelphia, treated erysipelas with pilocarpus.

I have had the opportuntiy of trying it in several cases, and I deem it to be my duty to report the result:

CASE I.—On the 2d of March, 1888, I was called to see J. R., who had a severe attack of erysipelas, affecting the right side of face, and spreading by the next day over the nose to left side of face and extending over the forehead. His eyes were badly swollen and had a profuse discharge from them. His pulse was quick and temperature up to 104° F. He was very weak, just recovering from a severe attack of catarrhal pneumonia. On account of his feeble condition I did not trust pilocarpus alone. Gave veratrum, quinine, iron, egg-nogg, milk-punch, when needed, and pilocarpus (fluid ext.) every three hours in 15 drop doses. Applied locally, salicylic acid, ʒss, glycerine and rose-water aa., ℥ss, and water ℥j. In two or three days the disease gave way. I did not know that pilocarpus had anything to do with checking the disease in this case.

CASE 2.—On the 20th of March I was called to see Mrs. J. She was similarly affected; face was so badly swollen that she could scarcely see, but had no discharge from her eyes. Gave ten grains of calomel combined with opium, quinine, veratrum, and applied locally the above preparation. On the 21st I found her some better. Gave muriate tinct. of iron in addition to veratrum and quinine. On the 22d I found her much worse, fever up to 103° F. I ordered pilocarpus (fluid ext.) in 15 drop doses every 3 hours in addition to veratrum and quinine. On the 23d I found her much better, and the disease had given way and she had no further trouble.

CASE 3.—On the 2d of April I was called to see Mrs. F. She was suffering with excruciating pains in the face and throat. The disease had attacked her face and spread downward. Before I was called a doctor had seen her and put her upon treatment.

I gave her morphine hypodermically to relieve pain, and a purgative, and ordered fluid ext. of pilocarpus in 15 drop doses every three hours.

I applied locally the above preparation. The disease gave way promptly, and she had no further trouble.

I will not worry you with details, but affirm that I have treated other cases, using nothing but a purgative, pilocarpus, and applied locally the salycylic preparation.

I am happy to say that I can control the disease much easier with pilocarpus than without it. I will not go into the *modus operandi*, but leave the explanation of the how to others.

